# Diffuse alveolar Hemorrhage as the initial presentation of systemic lupus erythematosus in a 57-year-old patient: a case report

**DOI:** 10.1093/omcr/omaf115

**Published:** 2025-07-27

**Authors:** Ahmed Abdulhussain Shahatta, Omar Radhwan Mohammed, Sajjad Ghanim Al-Badri, Muntadher Yousif Hasan Al Gehadi, Khadija Naeem, Aditya Duhan

**Affiliations:** College of Medicine, University of Thi-Qar, Thi-Qar, Iraq; Baghdad Teaching Hospital, Baghdad, Iraq; College of Medicine, University of Warith Al-Anbiyaa, Karbala, Iraq; University of Baghdad, College of Medicine, Baghdad, Iraq; Department of Medicine, Nishtar Hospital, Multan, Pakistan; Department of Radiology, SUNY Upstate Medical University, Syracuse, New York, United States

**Keywords:** autoimmune disease, systemic lupus erythematosus, diffuse alveolar hemorrhage

## Abstract

Systemic lupus erythematosus (SLE) is an autoimmune systemic disease that presents a diagnostic challenge due to a wide range of clinical manifestations affecting multiple organs. Particularly rare is its presentation with diffuse alveolar hemorrhage (DAH), a severe complication that arises from the disruption of the capillary-alveolar barrier and can rapidly progress to a life-threatening condition.

We report a case of a 57-year-old female who presented to the emergency department experiencing dyspnea and hemoptysis. Her initial evaluations revealed anemia and thrombocytopenia, with high-resolution computed tomography scans showing diffuse bilateral infiltrates indicative of DAH. The findings led to the diagnosis of previously unrecognized SLE. This case underscores the importance of considering SLE in the differential diagnosis of DAH, emphasizing the necessity for prompt diagnostic workup and aggressive treatment in such critical presentations to improve patient outcomes.

## Introduction

Systemic lupus erythematosus (SLE) is a complex autoimmune disorder with diverse clinical manifestations, making its diagnosis challenging, especially in atypical cases. Diffuse alveolar hemorrhage (DAH) is a rare but severe complication of SLE, characterized by intrapulmonary bleeding due to alveolar-capillary barrier disruption [1]. Diagnosing DAH in the context of SLE is further complicated by the need to distinguish it from other causes of pulmonary hemorrhage, such as infections or vasculitis, as management strategies vary significantly [2]. This case report discusses a 57-year-old woman with previously undiagnosed SLE, presenting with dyspnea and hemoptysis. HRCT findings confirmed DAH, emphasizing the importance of considering SLE in patients with acute respiratory distress and pulmonary hemorrhage for timely diagnosis and intervention.

## Case presentation

**Figure 1:**
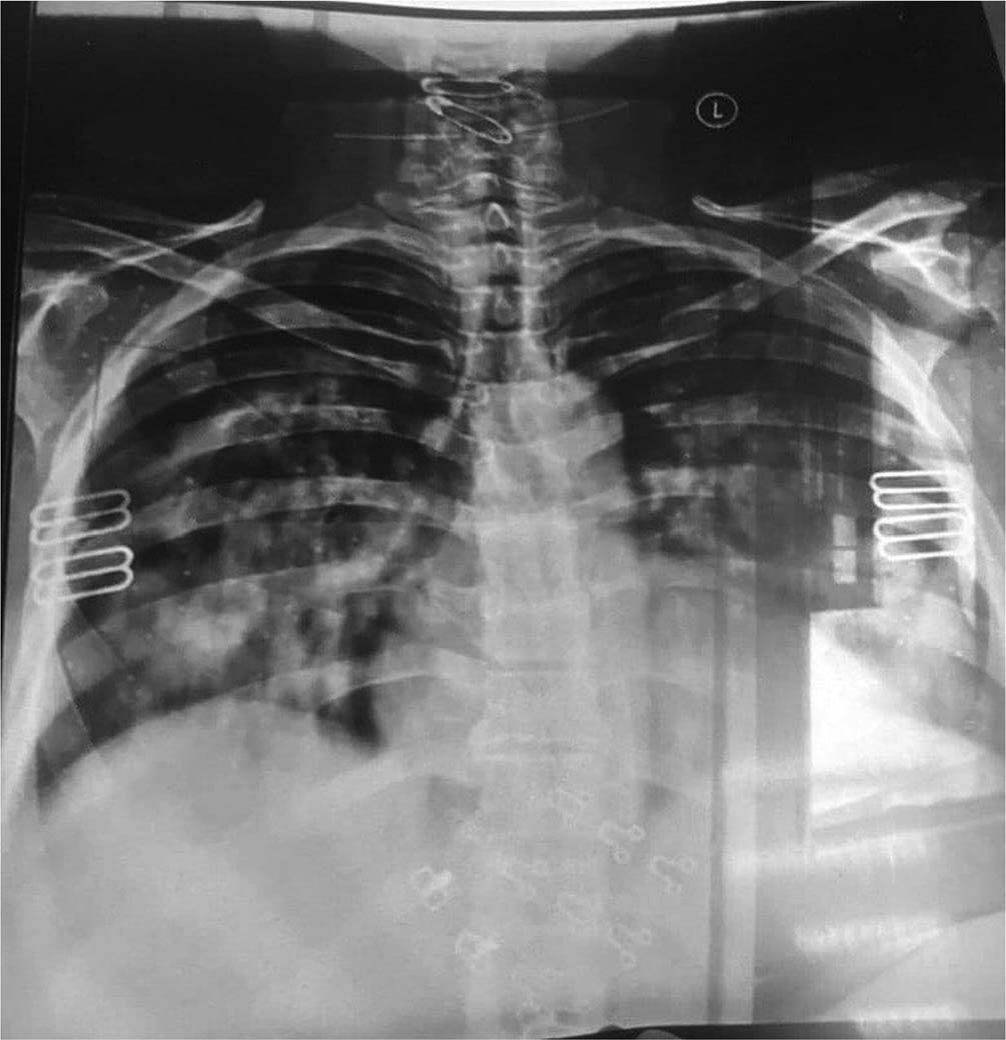
Chest radiograph shows: Bilateral diffuse infiltrates.

**Figure 2:**
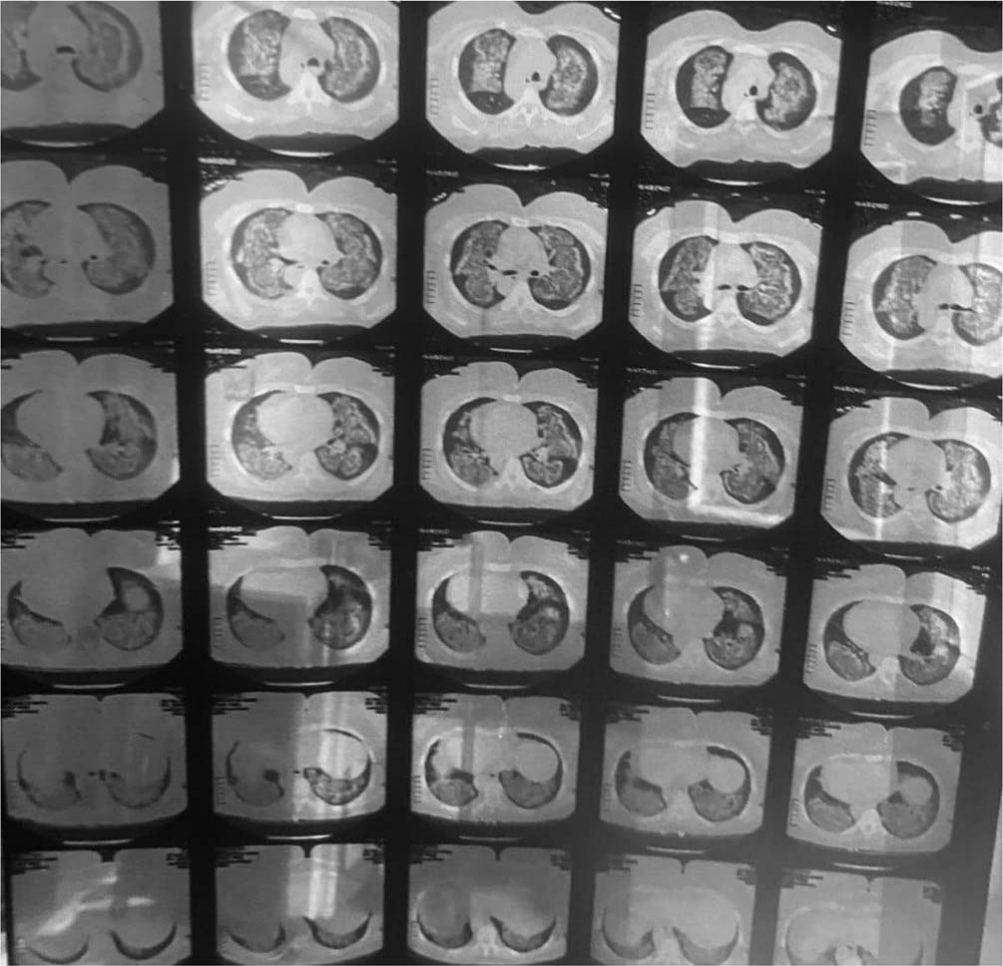
A high-resolution computed tomography (HRCT) of the lungs shows: Diffuse ground-glass opacities, which were highly suggestive of DAH.

A 57-year-old female, with a known case of hypertension, presented to the emergency department (ED) with a complaint of sudden onset shortness of breath and hemoptysis persisting for one day. This episode has occurred for the second time within one week. The patient reported a progressive deterioration characterized by an increased frequency of cough and worsening shortness of breath. The patient denied experiencing chest pain, palpitations, or a history of lower limb swelling, Additionally, the patient was not receiving any anticoagulant therapy and had no history of using other medications, including over-the-counter drugs, supplements, or herbal remedies. However, she had been prescribed an angiotensin receptor blocker (valsartan/amlodipine 160/10 mg), which had been initiated three months prior to her presentation. He denied a history of significant bleeding manifestations, such as gastrointestinal bleeding or spontaneous bruising. On admission, her physical examination was notable for diffuse coarse crepitations on her chest auscultation with the absence of peripheral edema, jugular venous distension, and abnormal heart sounds. Vital signs revealed a temperature of 36.7°C, blood pressure of 160/90 mmHg, and an oxygen saturation of 81% on room air. Subsequently, a chest radiograph was ordered and showed bilateral diffuse infiltrates (Fig. 1). A high-resolution computed tomography (HRCT) of the lungs revealed diffuse ground-glass opacities, which were highly suggestive of DAH (Fig. 2).

Laboratory investigations revealed a hemoglobin of 8.3 g/dl (normal range in females: 12–15 g/dl), as well as a platelet count of 3.7 × 10^3^/μl (normal range: 150–400 × 10^3^/μl) and white blood cell count of 4 × 10^3^/μl (normal range: 5–10 × 10^3^/μl). Creatinine of 2.1 mg/dl (normal range: 0.6–1.1 mg/dL for women) and Blood Urea Nitrogen (BUN) of 36 mg/dl (normal range: 7–20 mg/dl). Total Bilirubin of 2.8 mg/dl (normal range: 0.3–1.2 mg/dl), and Direct Bilirubin of 0.5 mg/dl (normal range: 0.0–0.3 mg/dl).

A workup for the underlying cause of this DAH was initiated; C-ANCA, p-ANCA, anti-GBM, anti-histone antibody, anti-Ro Ab, and anti-La Ab were negative. Anti-nuclear antibodies (ANA) at a titer of 6.0 index (normal range: < 1.00 negative, 1.00–1.20 borderline, > 1.20 positive). Additionally, anti-double strand DNA (anti-dsDNA) IgM was elevated at 22.30 U/ml (normal range: < 20.00 U/ml normal, > 20.00 U/ml elevated). C3 level was 0.71 g/L (normal: 0.80–1.85) and C4 level of 0.26 g/dl (normal: 0.10–0.40 g/dl). Reactive protein (CRP) was 55 mg/L (normal range: < 10 mg/l), and Erythrocyte Sedimentation Rate (ESR) was 70 mm/hr (normal range for females: 0–20 mm/hr). The patient underwent transthoracic echocardiography, which revealed only mild left ventricular hypertrophy with an interventricular septum thickness of 13 mm and normal left ventricular systolic function (ejection fraction: 63%). Additionally, NT-proBNP levels were 396 pg/ml. Sputum and blood cultures, Gram stain, acid-fast bacilli (AFB) stain, GeneXpert testing, and PCR for COVID-19 and influenza viruses were all negative. Finally, the renal biopsy demonstrated a mixed pattern of lupus nephritis, with predominant Class III LN changes and some features of Class II LN, according to the ISN-RPS classification.

According to EULAR/ACR criteria for SLE which has a sensitivity of 96.1% and specificity of 93.4%, the patient scored 15 and could increase to 25, with only 10 enough to be classified as SLE.

The patient was treated with a pulse dose of methylprednisolone (1 g a day) for four days, one dose of intravenous cyclophosphamide, and five days of plasmapheresis. After several days, the patient’s hemoptysis completely resolved, and hemoglobin levels stabilized. He was discharged in good clinical condition.

## Discussion

Systemic lupus erythematosus (SLE) primarily affects young women aged 15 to 45, with a female-to-male ratio of 9:1 [3]. Diagnosis in older adults, especially those over 50, is rare and presents unique challenges. A study of patients over 80 showed a mean diagnosis age of 84.3 years, with common symptoms including arthritis and skin involvement, but a lower incidence of renal complications [4]. This case is notable for the patient’s advanced age and severe presentation with diffuse alveolar hemorrhage (DAH).

Our patient, a 57-year-old female with hypertension, presented with shortness of breath, hemoptysis, and progressive respiratory deterioration. Clinical suspicion for DAH was confirmed by chest CT imaging.

In a multicenter study, 35 SLE patients with DAH showed a mortality rate of 40%, with hemoptysis being the most common symptom [4] Hematological abnormalities such as anemia and thrombocytopenia are frequent in SLE, often serving as initial manifestations caused by immune-mediated mechanisms. Positive antinuclear antibody (ANA) tests, elevated anti-dsDNA levels, and low complement C3 are critical indicators of SLE, with anti-dsDNA strongly associated with disease activity and lupus nephritis. [5] This case highlights the diagnostic challenges of SLE in older adults, where symptoms overlap with comorbidities like hypertension and kidney disease. Pulmonary involvement, particularly DAH, complicates diagnosis. Early recognition is crucial, and plasmapheresis is a critical treatment option when bronchoscopy is contraindicated [6].

Diffuse alveolar hemorrhage (DAH), which occurs in approximately 2%–5% of SLE patients, carries mortality rates ranging from 40%–60%, depending on the severity of the disease and the timeliness of intervention. The incidence of diffuse alveolar hemorrhage (DAH) in systemic lupus erythematosus (SLE) patients can vary significantly across different populations. A study involving 943 SLE Chinese patients reported a DAH frequency of 4.98%. [7] Another study involving 122 SLE Colombian patients, seven patients presented this complication (5.7%). [8] This case demonstrates the unusual presentation of DAH as the initial manifestation in an older adult with SLE. The disease frequently affects the pulmonary and renal systems, with lupus nephritis posing a significant risk [9]. Nearly half of SLE patients experience pulmonary involvement [10]. Pulmonary involvement in this case can mimic conditions like ARDS from infection or heart failure, which were ruled out through transthoracic echocardiography, NT-proBNP levels, and physical examination findings, including the absence of peripheral edema, jugular venous distension, and abnormal heart sounds. Negative microbiological tests further exclude infection as the primary cause. Diffuse alveolar hemorrhage (DAH) in systemic lupus erythematosus (SLE) is primarily caused by immune-mediated mechanisms, including immune complex deposition and complement cascade activation. DAH in SLE is often linked to autoantibody-mediated pulmonary capillaritis [11], while excessive complement activation contributes to endothelial injury and increased vascular permeability, allowing red blood cells to leak into the alveoli [12]. Late-onset SLE, as observed in this case, may reflect a unique immunological profile with delayed onset, complicating the diagnostic process. Thrombocytopenia is a well-known risk factor for bleeding, and it is plausible that it could contribute to the development of DAH in SLE patients. In this case, although the patient had thrombocytopenia, no significant hemorrhagic manifestations, such as gastrointestinal bleeding or spontaneous bruising, were observed. Thus, the mechanism of the pathological condition was more likely attributed to an immune response in SLE rather than thrombocytopenia. Drug-induced lupus (DIL) was considered in the differential diagnosis of late-onset SLE. However, a thorough review of the patient’s medication history was conducted to evaluate this possibility. The patient had been receiving valsartan/amlodipine (160/10 mg) for hypertension, initiated three months prior to symptom onset. There was no history of other medication use, including supplements, herbal remedies, or over-the-counter drugs. Furthermore, anti-histone antibody testing was negative, reducing the likelihood of DIL as the underlying etiology [13].

Regarding renal involvement in this patient, the significant elevation in serum creatinine cannot be solely attributed to Class II lupus nephritis, but the presence of active Class III disease, according to the ISN-RPS classification, can explain renal function deterioration [14]. Additionally, the patient had a history of hypertension and was on an angiotensin receptor blocker (valsartan/amlodipine 160/10 mg), which had been initiated three months prior to presentation. Given the absence of baseline or follow-up renal function tests, ACE inhibitor-related nephrotoxicity cannot be entirely excluded as a possible contributor to elevated renal indices.

Diagnosing SLE in older adults is challenging due to overlapping symptoms with age-related conditions like hypertension and chronic kidney disease. In this case, the absence of classical features made the diagnosis difficult, but adherence to the 2019 EULAR/ACR criteria supported the identification of SLE, While the criteria assist in the identification of cases, a comprehensive clinical assessment remains essential for a definitive diagnosis. Emphasizing the importance of systematic evaluation and early identification for timely treatment [15].

Management of DAH in SLE requires prompt and aggressive immunosuppressive therapy. Corticosteroids are the cornerstone of treatment, often combined with cyclophosphamide or rituximab in severe cases. [16] Therapeutic plasma exchange (TPE) is increasingly recognized for refractory or rapidly progressive cases, particularly in conditions like DAH, which occurs in approximately 30% of patients requiring intervention. [17] In this case, the coexisting lupus nephritis required a comprehensive immunosuppressive approach targeting both pulmonary and renal involvement. The patient’s clinical improvement highlights the importance of early diagnosis and timely treatment in managing life-threatening complications.

This report has limitations, including the lack of long-term follow-up, which restricts the understanding of sustained outcomes. As a single case, the findings may not be generalizable. Further investigations, such as bronchoscopy or additional autoimmune markers, could have provided more insights.

## Conclusion

This case highlights the importance of atypical SLE presentations, especially in older people, where symptoms like DAH and lupus nephritis can complicate the diagnosis. Early recognition, a thorough autoimmune workup, and timely immunosuppressive therapy were crucial in improving the patient’s outcome. This report also emphasizes the value of a multidisciplinary approach in effectively managing complex autoimmune disorders.
